# MAPK activation in mature cataract associated with Noonan syndrome

**DOI:** 10.1186/1471-2415-13-70

**Published:** 2013-11-12

**Authors:** Noriyasu Hashida, Xie Ping, Kohji Nishida

**Affiliations:** 1 Department of Ophthalmology, Osaka University Medical School, room E7, 2-2 Yamadaoka, Suita, Osaka 565-0871, Japan; 2 Department of Ophthalmology, the First Affiliated Hospital of Nanjing, Medical University, Nanjing, China

**Keywords:** Noonan syndrome, Ocular manifestation, Mitogen-activated protein kinase (MAPK), Cataract

## Abstract

**Background:**

Noonan syndrome is an autosomal, dominantly inherited disease; it is physically characterized by short stature, short neck, webbed neck, abnormal auricles, high arched palate, and cardiovascular malformation. Its pathological condition is thought to be due to a gain-of-function mutation in the Ras-mitogen-activated protein kinase (MAPK) signal transduction pathway. Eyelid abnormalities such as ocular hypertelorism and blepharoptosis are the most commonly observed eye complications.

**Case presentation:**

We report a case of Noonan syndrome associated with mature cataract that required operation. A 42-year-old man was diagnosed with Noonan syndrome at the age of 1 year. He underwent an eye examination after complaining of decreased visual acuity in the right eye and was diagnosed with mature cataract, which was treated by cataract surgery. There were no intraoperative complications, and the postoperative course was uneventful. Protein analysis of lens capsule and epithelium at capsulorhexis showed MAPK cascade proteins such as ERK and p38MAPK were upregulated. An abnormality in the *PTPN11* gene was also observed; a potential mechanism of cataract onset may be that opacity of the lens rapidly progressed due to abnormal activation of the Ras-MAPK signal transduction pathway.

**Conclusion:**

This case highlights the possible association of cataract formation with MAPK cascade protein upregulation in Noonan syndrome.

## Background

Noonan syndrome (NS, OMIM 163950) is a common genetic disorder characterized by congenital heart disease, short stature, thoracic abnormality, cryptorchidism, mental retardation, and a typical facial appearance, among other characteristics [1,2]. NS is transmitted as an autosomal dominant trait, and is genetically heterogeneous. Originally, Tartaglia et al. identified missense mutation in *PTPN11*, which encode Src homology 2-domain phosphatase 2 (SHP-2) in 50% of individuals with NS [3]. Gain-of-function mutations in the PTPN11 gene lead to activation of the Ras-mitogen-activated protein kinase (MAPK) signal transduction pathway. Loss-of function or dominant negative mutations in *PTPN11* have been also documented in individual in NS with multiple lentigines (NS/ML, referred to as LEOPARD syndrome (LS; OMIM 151100)) [4]. So far, heterozygous mutations in nine genes (*PTPN11, KRAS, NRAS, SOS1, RAF1, BRAF, SHOC2, MEK1,* and *CBL*) in the Ras-MAPK signaling pathway cause NS or closely related conditions, comprising LS, Noonan-like syndrome with loose anagen hair (NS/LAH, OMIM 607721), and the recently documented “CBL mutation associated” syndrome [5,6]. Noonan-like syndromes comprising Costello syndrome (CS; OMIM 218040), cardiofaciocutaneous syndrome (CFCS, OMIM 115150), neurofibromatosis type 1 (NF1; OMIM 162200), and Legius syndrome (NFLS, OMIM 611431) are disorders clinically related to NS and also harbor mutations in *HRAS, KRAS,NF1, BRAF, SPRED1, MEK1,* and *MEK2* genes [5,6]. These genes are involved in several developmental processes that control morphology determination, organogenesis, synaptic plasticity, and growth [3-7]. Based on this shared pathogenetic mechanism and clinical overlap, these diseases have been grouped into a single family, which has been termed the Ras-MAPK syndrome, alternatively the RAS-opathies.

Ocular hypertelorism, blepharoptosis, strabismus, ametropia, and cataract have been reported as eye complications in NS [8]. However, the mechanisms of onset of cataract in this order are not clear, and only one case requiring operation has been reported previously [9]. We reported a case of NS associated with mature cataract that required operation. We could also prove MAPK activation and suggested the possible association of MAPK cascade with cataract formations in this disease.

## Materials and methods

### Subjects

The Institutional Review Board of the Osaka University Medical School approved the research protocol, and all patients provided informed consent. This study was registered with the approval of Institutional Review Board of the Osaka University Medical School. In the protein analysis, the lens capsule and epithelium at capsulorhexis of the patient with Noonan syndrome or those of senile cataract patient (as control) were collected during surgery.

### Western blot analysis of p38α MAP Kinase and ERK phosphorylation

After centrifuge, the sediment homogenized in a blender taken in RIPA buffer (R0278, Sigma-Aldrich, Tokyo, Japan) supplemented with 1% Protease inhibitor cocktail (P8340, Sigma-Aldrich) at 4°C. Lysates were placed on ice for 15 minutes, and centrifuged at 14,000 rpm for 10 minutes at 4°C. The supernatants were collected and preserved at -70°C. Protein concentrations were determined by Coomassie Bradford Protein Assay Kit (Catalog No. 23200, Thermo Fisher Scientific Inc., IL, USA). 13 μg of the total protein per sample was diluted with Laemmli Sample Buffer (Catalog No.161-0737, Bio-Rad, CA, USA), heated at 95°C for 4 min, separated by SDS-PAGE (Multigel II Mini, Cosmo Bio, Tokyo, Japan), and electroblotted onto polyvinylidene fluoride membrane (PVDF, GE Healthcare, Buckinghamshire, UK). After blocking with 2.5% skim milk for 1 hour at room temperature, the membranes were incubated with a p38α MAP Kinase rabbit monoclonal antibody (1:1000, Catalog No.2371, Cell Signaling, Danvers, MA, USA), a rabbit polyclonal anti-phospho-extracellular signal-regulated kinase (ERK) antibody (1:2000, Catalog No.4370, Cell Signaling), a rabbit polyclonal anti-ERK to detect total ERK protein (1:1000, Catalog No.4695, Cell Signaling), or anti-GAPDH (14C10) (1:2000, Catalog No.2118, Cell Signaling) over night at 4°C. After washing with 0.1% Tris-buffered saline (TBS)-Tween, blots were incubated with horseradish peroxidase (HRP)-conjugated goat anti-rabbit IgG (1:2500, Catalog No.7074, Cell Signaling) for 1 h at room temperature. The blots were then washed three times with 0.1% TBS-Tween and the signals were visualized by an ECL kit (GE Healthcare, Buckinghamshire, UK) according to the manufacturer’s protocol. The densities of immunoreactive bands were measured using Image J for Windows (NIH, Bethesda, MD, USA).

## Case presentation

**Figure 1:**
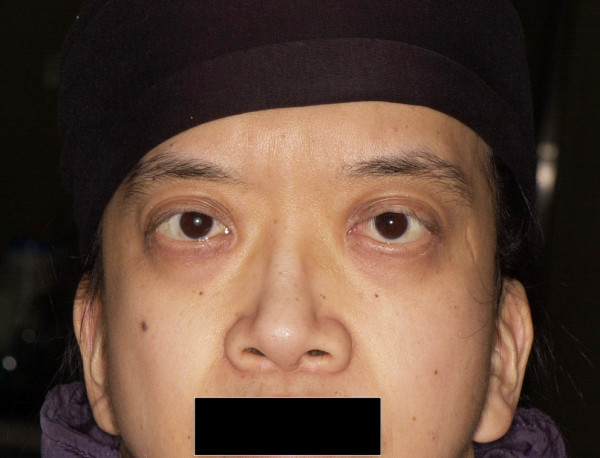
**Characteristic facial appearance.** Patient presents with typical appearance such as exophthalmos and abnormal auricles.

**Figure 2:**
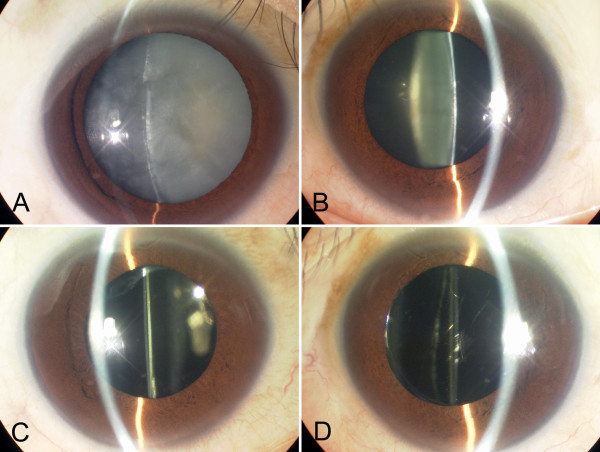
**Slit lamp examination on initial before and after the surgery.** Initial slit lamp examination revealed **(A)** mature cataract in the right eye **(A)** and mild cataract in the left eye **(B)**. **(A)** Mature cataract was observed in the right eye. Slit lamp examination after the cataract surgery in the right eye **(C)** and the left eye **(D)**, showing no complication.

**Figure 3:**
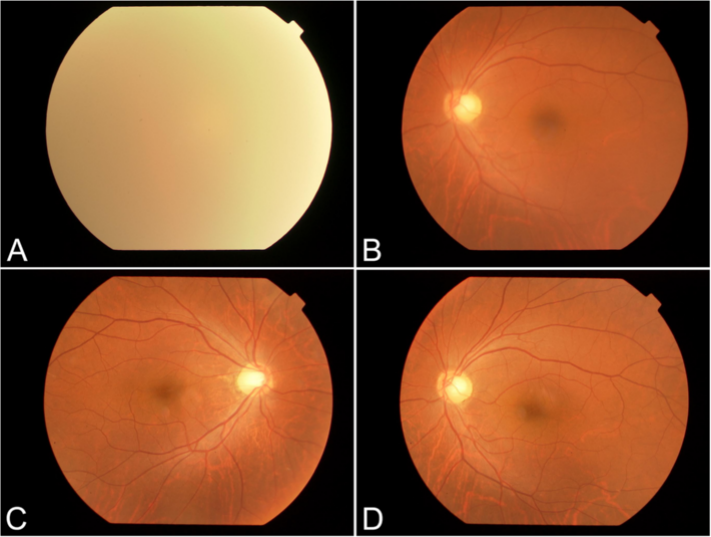
**Fundus photograph before and after the surgery. (A)** Fundus photograph of the right eye, but fundus could not be seen due to mature cataract. **(B)** Fundus photograph of the left eye, showing normal appearance. Recent fundus photograph after the cataract surgery in the right eye **(C)** and the left eye **(D)**, showing normal appearances.

**Figure 4:**
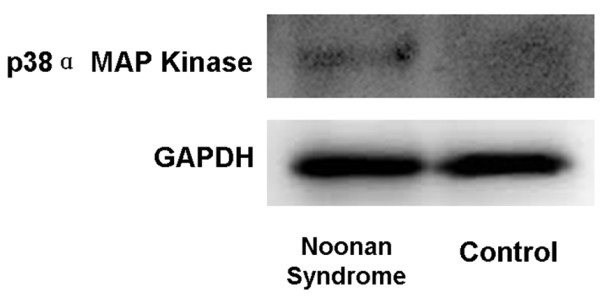
**Western blot analysis of p38α MAP Kinase.** Western blot of p38α MAP Kinase in lens capsule and epithelium at capsulorhexis from the patient with Noonan syndrome and senile cataract (control). Blot probed with the anti- p38α MAP Kinase antibody was detected in lens lysates from the patient with Noonan syndrome, but not from the patient with senile cataract (control).

**Figure 5:**
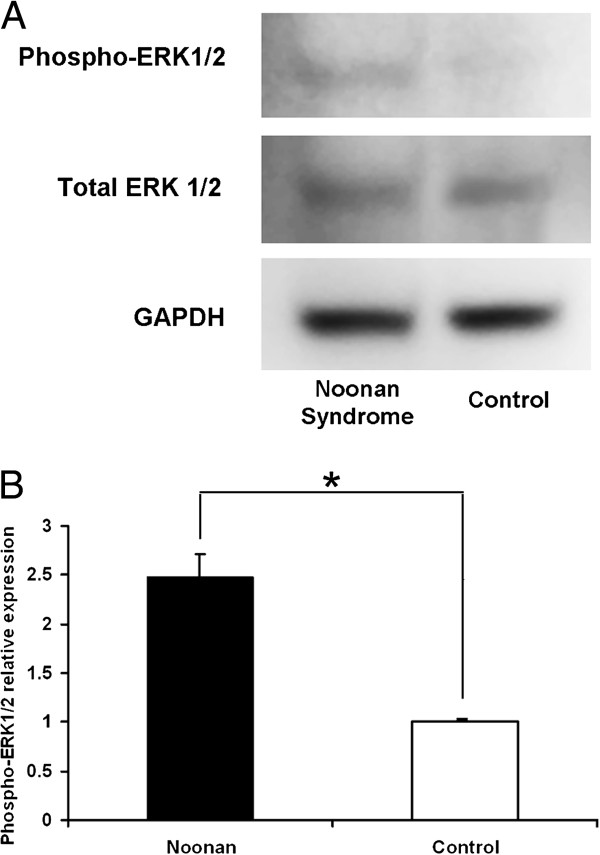
**Western blot analysis of phosphorylated ERK.** Phosphorylated extracellular signal-regulated kinase (p-ERK1/2) expression in Western blot. **(A)** A representative blot. p-ERK expression in lens lysates from the patient with Noonan syndrome and senile cataract (control). Western blot analysis revealed that p-ERK expression increased in lens lysates from the patient with Noonan syndrome. **(B)** Semi-quantitative analysis of the band intensity showed an increase in relative p-ERK expression (values normalized to total ERK expression) in lens lysates from the patient with Noonan syndrome compared with that from control (*n* = 6, *P < 0.001).

A 42-year-old man experienced decreased visual acuity in the right eye before 2 years, but did not seek treatment. Later, he became aware of severely decreased visual acuity in the right eye several months ago and visited our hospital. The patient was born at 37 weeks of gestation with a weight of 2,300 g and had neonatal asphyxia at birth and had severe jaundice in neonatal period. The patient has a high arched palate, webbed neck, short neck, ocular hypertelorism, exophthalmos, and dilated cardiomyopathy. These factors led to a clinical diagnosis of NS at the age of 1 year (Figure 1). Cardiac disease is controlled through the use of oral drugs. Without receiving growth hormone treatments, his final body height was 154 cm, which is below the potential average height of Japanese men. Initial eye examination suggested that his corrected visual acuity was 0.06 in the right eye and 0.9 in the left with normal intraocular pressure. His corneas and irises showed no abnormalities and no anterior chamber inflammation was observed. Both eyes showed cataracts, and mature cataract was observed in the right eye (Figure 2A, B). The patient had no history of traumatic eye injuries or self-injurious behavior. The left eye fundus showed no abnormal findings (Figure 3A, B). Although the right eye fundus could not be seen, B-mode ultrasonography showed no abnormal findings. No abnormal pupillary light reflexes were observed. In addition to the mature cataract in the right eye, the cataract in the left eye progressed rapidly during the follow-up period, leading to decreased visual acuity. Cataract surgery was indicated for both eyes, and it was performed with no intraoperative complications (Figure 2C, D). He experienced no postoperative intraocular pressure elevation, infection, or delayed inflammation and there were no postoperative abnormalities in the right eye fundus (Figure 3C, D). Genetic analysis revealed a mutation in the *PTPN11* gene. The identified PTPN11 mutations (c.188A > G [p.Tyr63Cys]) encode alterations located in the N-terminus of SH2 (Src homology 2) domain, catalytic domain in PTPN11. Protein analysis also showed that the expression of p38α MAP kinase was detected in lens lysates from the patient with NS but, the expression of p38α MAP kinase could not be detected in patient with senile cataract (control), (Figure 4). And also, the p-ERK1/2 expressed in a very low level in lens lysates from the patient with senile cataract (control), while in the patient with NS, it was significantly increased (n = 6, *P* <0.001) (Figure 5). His postoperative corrected visual acuity was 1.2 in the right eye and 1.0 in the left, and the postoperative course was uneventful.

## Discussion

NS shows physical characteristics similar to those of Turner syndrome, but occurs in both men and women with normal karyotypes and is an independent disease entity from Turner syndrome. Because NS has no explicit diagnostic criteria, it is diagnosed on the basis of its clinical manifestations by using a scoring system [10]. van der Burgt score is usually used for the diagnosis of NS. This scoring system contains 6 categories with 2 alternatives in each category [11]. The syndrome is systemically characterized by short stature, short neck, webbed neck, auricular abnormalities, high arched palate, and cardiovascular malformation, among others. Our patient showed nearly all systemic abnormalities and was diagnosed with definite NS during his childhood. Although ocular hypertelorism, epicanthus, thick eyelids, palpebral ptosis, and strabismus are the primary eye symptoms observed in NS [8], there has been only a report of cataract complications required operation [9]. Thus, we reported, for the first time, local activation of MAPK signaling pathway in lens of NS patient.

The MAPK family consists of three subfamilies, *such as* extracellular signal-regulated kinase (ERK) 1 and 2, the c-Jun N-terminal kinase (JNK, also known as the stress-activated protein kinase), and p38 MAPK. The classic MAPKs, ERKs, are principally activated in response to growth factors, whereas JNK and p38 are activated by various stresses, including TNF-α treatment, UV light, x-ray irradiation, heat-shock, and H_2_O_2_ treatment [12]. According to the previous reports, transforming growth factor-β (TGF-β) and basic fibroblast growth factor are crucial factors for lens physiology [13-15]. Binding of these factors to lens cell surface receptors initiate cell signaling pathways that include MAPK/ERK signal transduction pathway. The RAS/RAF/MEK/ERK pathway is the classical RAS-MAPK signaling pathway implicated in growth-factor mediated cell proliferation, differentiation, and death [16]. In normal state, appropriate expression of TGF-β is important for ERK activation and physiological cataract formation. The Ras-MAPK pathway is reportedly involved in lens differentiation and maturation [17-19].

We proved MAPK activation in the lens and suggested the possible association of MAPK signaling cascade with cataract formation in cataract with NS. Lee NB et al. reported cataract occurs in 8% of NS [8]. In a study of congenital heart disease, 3 cases (1.3%) out of 240 had NS, and congenital cataract was present in six cases (2.5%) [20], suggesting that the RAS-opathies, a group of disorders with RAS-MAPK signaling pathway and overlapping neuro-cardio-facial-cutaneous phenotypes, play roles in the possible pathogenesis of this disease. It is well known that Down syndrome and Turner syndrome cause cataract as an eye complication. In contrast, NS shows an overwhelmingly high incidence of corneal and eyelid abnormalities; although cataract complications are observed in this disease, the incidence is lower than that found in Down syndrome [8]. Considering the involvement of MAPK signaling pathway for the cataract formation, genetic analysis revealed a mutation of *PTPN11* gene in our patient. The *PTPN11* gene is located in the upstream portion of the Ras-MAPK pathway and is thought to be involved in cell growth, differentiation, and senescence [3,21]. The cataract may have been observed during childhood, but lens opacity may have increased in severity with aging and accumulation of metabolic abnormalities, since no abnormalities were observed in other pathways. It is likely that gain of function of MAPK signaling pathway in NS patients directly activated ERK irrespective of TGF-β upregulation and led to advanced cataract formation.

## Conclusions

We report a case of mature cataract complicated by NS and found the local activation of MAPK signaling pathway for cataract formation. Although lens opacity varies in severity, it can progress during the course of the syndrome. The current case highlights the fact that periodic observations of the lens opacities are necessary in patient with NS.

## Informed consent

Written consent was obtained from the patient for publication of this material. A copy of the consent is available for review.

## Competing interests

The authors declare that they have no competing interests.

## Authors’ contributions

NH: patient interaction and diagnosis, Western blot experiment, drafting of manuscript, final approval of manuscript, XP: patient diagnosis, Western blots experiment, final approval of manuscript, KN: patient interaction and diagnosis, final approval of manuscript. All authors read and approved the final manuscript.

## Pre-publication history

The pre-publication history for this paper can be accessed here:

http://www.biomedcentral.com/1471-2415/13/70/prepub
